# Height of the foot longitudinal arch and anterior cruciate ligament injuries

**DOI:** 10.1590/1413-78522014220600659

**Published:** 2014

**Authors:** Paulo César de César, Jairo André de Oliveira Alves, João Luiz Ellera Gomes

**Affiliations:** 1. Universidade Federal do Rio Grande do Sul, Faculdade de Medicina, Porto Alegre, RS, Brazil, Faculdade de Medicina da Universidade Federal do Rio Grande do Sul, Porto Alegre, RS, Brazil

**Keywords:** Foot, Anterior cruciate ligament/injuries, Anthropometry

## Abstract

**OBJECTIVE:**

To evaluate the association between the height of the medial longitudinal arch of the foot and non-contact injuries of the anterior cruciate ligament.

**METHODS:**

One hundred and five patients were included in this case-control study. The case group consisted of 52 patients with non-contact injury of the anterior cruciate ligament. Fifty-three individuals with no history of symptoms regarding to feet or knees comprised the control group. An anthropometric assessment of the bony arch index was performed, which consisted of measuring the ratio of the height between the navicular bone to the ground and the distance from the most posterior support point of the calcaneus to the first metatarsal-phalangeal joint. Gender, height, weight, body mass index and the frequency of sports practice were also evaluated.

**RESULTS:**

Subjects in the case group had significantly higher medial longitudinal arches than individuals in the control group.

**CONCLUSION:**

Individuals with rupture of the anterior cruciate ligament had higher arches than the corresponding controls, suggesting an association between a high medial longitudinal arch of the foot and injury of the anterior cruciate ligament. **Level of Evidence III, Case-Control Study**

## INTRODUCTION

The modification of the foot with the development of the medial longitudinal arch (MLA) was an important evolutionary milestone that allowed humans to walk on the floor instead of on trees.^1^^,^ 2 MLA provides better support for the weight of the body during the stance phase of the gait cycle as it enhances the action of the plantar flexor muscles^1^^,^ 3 and allows dissipation of impact in bipedal motion.^4^

The search for an association between the height of the medial longitudinal arch and the incidence of injury during sports practice or military training has been the subject of several studies.^5^^-^ 11

In the present study, we evaluated the possible association between injuries by non-contact of the anterior cruciate ligament (ACL) of the knee and the height of the medial longitudinal arch of the foot.

The ACL rupture is a common injury in orthopedic practice, occurring at a rate of one new case per 3,000 individuals each year.^12^ This high frequency is one of the factors that has motivated the study of this injury and attempts at correction with MLA measurements. Since 70% of ACL injuries are due to a non-contact event^13^ - i.e., the injury develops without any direct trauma, intrinsic and extrinsic factors are possibly associated with ACL injury.

One point of controversy is the technique used to measure MLA. Several methods are available, such as footprints,^14^^,^ 15 radiographic evaluation of the foot^16^ and anthropometric assessment of the foot.^5^ Surprisingly, the comparison between these different forms of measurement of MLA shows little agreement,^17^^,^ 18 which creates a challenge about which method should be chosen. In our study, we chose to use the anthropometric assessment, which seemed to better reflect the measurement of MLA.

According to our experience, this is the first study that attempts to match the height of the medial longitudinal arch of the foot with non-contact injuries of the anterior cruciate ligament of the knee.

## MATERIALS AND METHODS

This was a case-control study. The case group consisted of 52 patients and the control group of 53 individuals. The cases were selected from the Knee Treatment Group of our Service and our controls were randomly recruited from the various subspecialty groups in our institution. Inclusion criteria for the study group were: diagnosis of ACL rupture confirmed by physical examination, MRI and surgical findings, and a negative history of contact that could have caused the injury. The subjects in the case and control groups should not have had previous complaints about their feet or ankles. The age of cases and control subjects ranged between 18-40 years old.

**Figure 1:**
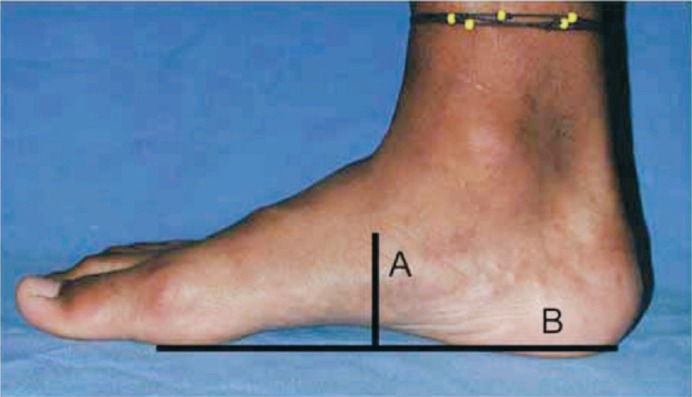
A) Height of the navicular bone from the floor; B) Foot length.

The selected method to measure the height of MLA was the anthropometric technique described by Cowan *et al*.^5^ The bony arch index was evaluated (Figure 1), which is the ratio between the height of the navicular bone from the ground and the length of the foot (distance between the most rear load support portion from the calcaneus to the first metatarsophalangeal joint). Measurements were obtained with each individual standing on a podoscope. In the case group, the ipsilateral foot was evaluated. In the control group, both feet were measured and the average between them was calculated. Both cases and controls were evaluated for gender, height, weight and weekly frequency of sports practice. All participants provided an Informed Consent Form before participating in the study, which was approved by the Ethics Committee of Hospital de Clinicas de Porto Alegre, Porto Alegre, RS, Brazil.

## RESULTS

All statistical analyzes were performed using Statistical Package for Social Sciences (SPSS Inc., Chicago, IL). The alpha value was set at 0.05. The variable gender was assessed using the chi-square test with Yates correction, while weight, height, relationship between navicular height and foot length and body mass index (BMI) were assessed by Student's t-test for independent variables. The variable "frequency of sports practice" was assessed by the Mann-Whitney test.

The analysis of gender showed 49 (94.2%) male patients in the case group and 44 (83%) males in the control group (p = 0.134). There were no significant differences between groups in height; the average was 1.76m in the case group, with a standard deviation (SD) of 0.07m, and 1.75 ± 0.1 m in the control group (p = 0.666). The average weight in the case group was 75.8 ± 9.7 kg, compared with 74.2 ± 13.8 kg in the control group (p = 0.509). The mean BMI was 24.4 ± 2.5 kg/m² and 24.0 ± 3.3 kg/m² in the case and control groups, respectively (p = 0.498). The average frequency of sports practice was three times per week in the case group, which ranged from one to four, and twice a week in the control group, which ranged between 1 and 5 (p = 0.203). The average bony arch index was 0.285 ± 0.053 in the case group and 0.262 ± 0.043 in the control group (p = 0.018); this was the only statistically significant difference between groups. The standardized effect size was 0.48 (95% confidence interval, 0.09 to 0.86), which corresponds to a small magnitude. The results are summarized in Table 1.

**Table 1:** Results

Variable	Case group (n = 52)	Control group (n=53)	p value
Gender (M/F)	49 (94.2%) / 3 (5.8%)	44 (83%) / 9 (17%)	p = 0.134
Heigh (m)	1.76 ± 0.07	1.75 ± 0.1	p = 0.666
Weight (kg)	75.8 ± 9.7	74.2 ± 13.8	p = 0.509
BMI (kg/m^2^)	24.4 ± 2.5	24.0 ± 3.3	p = 0.498
Frequency of sports practice (times per week)	3 (1 a 4)	2 (1 a 5)	p = 0.203
Bony arch index	0.285 ± 0.053	0.262 ± 0.043	p = 0.018

## DISCUSSION

As the feet are the foundation of the body, it is logical to assume that the forces transmitted through them during gait and sports practice will influence the incidence of injuries. In a study with 180 runners, James *et al*.^19^ found 232 musculoskeletal injuries and concluded that a foot with high arches is not a foot adapted for running. Giladi *et al*.,^7^ in a study with 295 recruits of the Israeli army, found a higher incidence of stress fractures of the lower limbs in people with high arches compared to those with flat arches (p <0.05). Kaufman *et al*.^20^ reported that individuals both with high and flat arches, at static and dynamic evaluation, showed an approximately two-fold higher incidence of stress fractures when compared to subjects with normal MLA. In this study, there was no association between the mobility of the ankle or subtalar joint and risk of stress fractures in the lower limbs. Cowan *et al*.^5 ^reported that the flat foot has an *odds ratio* (OR) of 1.0 for exercise-induced injuries against 3.0 for normal arch feet and 6.1 for feet with high arches. Therefore, in this study, there was an increasing trend in risk of injury by increasing the height of the MLA. Simkin *et a*l.^11^ reported that stress fractures of the femur and tibia are more common in patients with high arches, while stress fractures of the metatarsals are more common in feet with flat arches. Mei-Dan *et al*.^15^ retrospectively and prospectively evaluated 83 female recruits of the Israeli army and found that flat arcs are a risk factor for ankle sprain in retrospective evaluation, while in the prospective evaluation, despite a detectable trend, there was no statistical confirmation for flat arc as a risk factor for ankle sprain. Even though these studies have previously found an association between the height of MLA and injuries, others have not found such association.^6^^,^ 8 By analyzing the above items, we realized that there is an attempt to identify a potential association between the morphology of the arch and incidence of injuries during physical activity.

In our study, we found an association between the height of MLA and disruption by non-contact of the ACL, since the case group had a higher average arch index than the control group (p = 0.018). It should be noticed that there was no statistical difference between the groups in any of the other measured variables (weight, height, body mass index and frequency of sports practice), thus, reducing the potential for confounding factors. As previously mentioned, a high medial longitudinal arch has been associated with a number of injuries during physical activity. The same association was also demonstrated in our study, specifically regarding non-contact injuries of the anterior cruciate ligament. Approximately 70% of ACL injuries occur without physical contact,^13^ i.e., no history of direct trauma that could have played a causal role in the event, which leads to the assumption that intrinsic factors are associated with the pathogenesis of ACL injuries. Some of these factors have been identified, such as small width of the femoral intercondylar notch.^21^ Therefore, our study may be indicative of the existence of another intrinsic factor associated with the pathogenesis of LCA injury.

Despite the need for further research, we suggest two possible explanations for the association between MLA height and ACL injury. Feet with high arches have a reduced load support area reduced, which can modify the transmission of forces through the lower limbs, leading to increased stress on the ACL and subsequent injury. In this case, the ACL injury would be a stress lesion - i.e., a repetitive stress injury that exceeds the capacity for regeneration of the human body. Lentell *et al*.^22^ reported that individuals with chronic ankle instability have diminished proprioception. A similar change in proprioception may be present in patients with knee sprains and subsequent ACL injury, suggesting that proprioceptive changes are related to the morphology of the MLA.

A limitation of this study is the choice of method for MLA measurement, since there is disagreement in the literature when different evaluation methods are compared. A widely used method is the registration of plant footprint; however, its usefulness has been questioned. Cobey and Sella^17^ found a weak correlation in comparison of plantar footprints with radiographic evaluation of MLA. Hawes *et al*.^18^ also found a weak correlation between the plant footprint and direct clinical evaluation of MLA. In our study, we used a method of anthropometric evaluation, which consisted of measuring the ratio between the height of the navicular bone from the floor and the foot length (distance between the most rear load support portion of the calcaneus to the first metatarsophalangeal joint), called the bony arch index. According to previous studies, after evaluation of several multivariate analysis criteria, the bony arch index correlates better with injury during physical activity.^5^ Despite the evidence found in this group of patients, further studies with larger athletes samples are needed to make definitive conclusions about the influence of morphology of MLA on non-contact ACL injuries.

## CONCLUSION

In the present study we found an association between feet with high medial longitudinal arches and a higher incidence of non-contact ACL injuries, suggesting that high MLA can play a role as a risk factor for such ligament injuries.
